# A cost-effectiveness analysis of an integrated clinical-radiogenomic screening program for the identification of BRCA 1/2 carriers (e-PROBE study)

**DOI:** 10.1038/s41598-023-51031-1

**Published:** 2024-01-09

**Authors:** A. Di Pilla, C. Nero, M. L. Specchia, F. Ciccarone, L. Boldrini, J. Lenkowicz, B. Alberghetti, A. Fagotti, A. C. Testa, V. Valentini, E. Sala, G. Scambia

**Affiliations:** 1 Dipartimento di Scienze della Vita e Sanità Pubblica - Sezione di Igiene, Università Cattolica del Sacro Cuore, Rome, Italy; 2 UOC Ginecologia Oncologica, Dipartimento per le Scienze della salute della donna, del Bambino e di sanità pubblica, Fondazione Policlinico Universitario A. Gemelli IRCCS, Rome, Italy; 3 Università Cattolica del Sacro Cuore, Rome, Italy; 4 Fondazione Policlinico Universitario Agostino Gemelli IRCCS, Radiomics Research Core Facility, Gemelli Science and Technology Park, Rome, Italy; 5 UOC Radiologia, Diagnostica per Immagini, Radioterapia Oncologica ed Ematologia, Fondazione Policlinico Universitario A. Gemelli IRCCS, Rome, Italy

**Keywords:** Health care economics, Cancer prevention, Medical genomics

## Abstract

Current approach to identify BRCA 1/2 carriers in the general population is ineffective as most of the carriers remain undiagnosed. Radiomics is an emerging tool for large scale quantitative analysis of features from standard diagnostic imaging and has been applied also to identify gene mutational status. The objective of this study was to evaluate the clinical and economic impact of integrating a radiogenomics model with clinical and family history data in identifying BRCA mutation carriers in the general population. This cost-effective analysis compares three different approaches to women selection for BRCA testing: established clinical criteria/family history (*model 1*); established clinical criteria/family history and the currently available radiogenomic model (49% sensitivity and 87% specificity) based on ultrasound images (*model 2*); same approach used in model 2 but simulating an improvement of the performances of the radiogenomic model (80% sensitivity and 95% specificity) (*model 3*). All models were trained with literature data. Direct costs were calculated according to the rates currently used in Italy. The analysis was performed simulating different scenarios on the generation of 18-year-old girls in Italy (274,000 people). The main outcome was to identify the most effective model comparing the number of years of BRCA-cancer healthy life expectancy (HLYs). An incremental cost-effectiveness ratio (ICER) was also derived to determine the cost in order to increase BRCA carriers-healthy life span by 1 year. Compared to *model 1*, *model 2* increases the detection rate of BRCA carriers by 41.8%, reduces the rate of BRCA-related cancers by 23.7%, generating over a 62-year observation period a cost increase by 2.51 €/Year/Person. Moreover, *model 3* further increases BRCA carriers detection (+ 68.3%) and decrease in BRCA-related cancers (− 38.4%) is observed compared to *model 1*. *Model 3* increases costs by 0.7 €/Year/Person. After one generation, the estimated ICER in the general population amounts to about 3800€ and 653€ in *model 2* and *model 3* respectively. *Model 2* has a massive effect after only one generation in detecting carriers in the general population with only a small cost increment. The clinical impact is limited mainly due to the current low acceptance rate of risk-reducing surgeries. Further multicentric studies are required before implementing the integrated clinical-radiogenomic model in clinical practice.

## Introduction

Women who carry BRCA 1/2 genes pathogenic or likely pathogenic variants (BRCA 1/2 PV) have a cumulative risk of 61–79% and 11–53% for developing breast or ovarian cancer respectively by age 80^1,2^. The prevalence of BRCA1/2 PV carriers in the general population is around 0.67%^3^. Mutation identification enables women to opt for risk-reducing salpingo-oophorectomy (RRSO) in order to reduce ovarian cancer risk by 79–96%^4^ and risk-reducing mastectomy (RRM, with or without RRSO) to reduce breast cancer risk by 90–95%^5^.

Current indications for BRCA testing rely on established clinical criteria/family history based on priori BRCA probability thresholds to identify high-risk individuals^6,7^: in most countries, probability thresholds is set at 10%^8^. However, those criteria miss a large proportion of carriers who can benefit from screening/prevention^9,10^. Data regarding the cost-effectiveness of a population-based BRCA mutation screening are still controversial^11,12^ but there is increasing awareness of its benefit in maximizing early detection and cancer prevention. The development of new, cost-effective strategies for improving BRCA-related cancers prevention has therefore become an impelling health and social need.

The application of automated quantitative analysis techniques to bioimages and its association with biological and clinical endpoints has led to a novel approach called radiomics^13,14^. Obtaining quantitative information from imaging that goes beyond images themselves and the correlation between imaging phenotype and gene expression represents an innovative and very promising branch, called “radiogenomics”^15^. We hypothesize that the detection of BRCA 1/2 PV carriers through the radiomic analysis of US images of normal ovaries integrated with genomics data could represent a cost-effective, highly reproducible, large-scale extendable and time saving tool of cancer prevention.

Our group already showed the feasibility of developing an automated machine learning radiogenomics model with encouraging performances (49% sensitivity and 87% specificity) to identify BRCA status based on real world retrospectively collected US images of healthy ovaries acquired on different US machines^16^.

We hypothesize that the integration of such model together with current clinical/family history criteria to select patients for BRCA testing could result in a significantly higher detection of BRCA carriers, reducing overall BRCA-related cancers incidence due to prophylactic surgeries and increasing early cancer diagnosis due to properly planned follow-up. This aspect is particularly relevant for ovarian cancer since no validated screening programs are available and the overall prognosis remains poor^17^. Finally, over time cascade testing is expected to guarantee access to genetic testing to all carriers based only on anamnestic information.

To support these hypotheses, we conducted a cost-effective analysis comparing different models for the identification of BRCA1/2 carriers in the general population in Italy.

## Methods

### Model description

A model based on subsequent events related to the effects of BRCA1/2 genetic testing strategy in clinical practice was developed. The model starts with the identification of those women who may benefit from genetic testing and those who may *not*.

Women undergoing genetic testing who test positive are offered risk-reducing surgery options (it was acknowledged that BRCA 1/2 carriers of childbearing age could wait until the age of 35–45 years to undergo RRM/RRSO^18,19^). Positive testing also implies cascade testing of first-degree relatives from the age of 18.

In accordance with published data, a certain proportion of BRCA1/2 PV carriers will develop BRCA-related gynaecological cancers (in some cases, despite prophylactic surgery) loosing years of healthy life and increasing costs to the health care system. The number of BRCA-related cancer patients will then include both aware carriers before the onset of cancer (thus having had the option to opt for a possible risk-reduction strategy) and carriers not aware of their status until cancer onset.

To women who test negative, standard female cancer screening programme is offered that includes biannual mammogram, breast ultrasound, and annual gynaecological examination including pelvic ultrasound^20^.

Given the benefit from risk-reducing surgery, the long-term outcomes and costs (including follow-up ones) were considered for women testing positive only.

**Figure 1:**
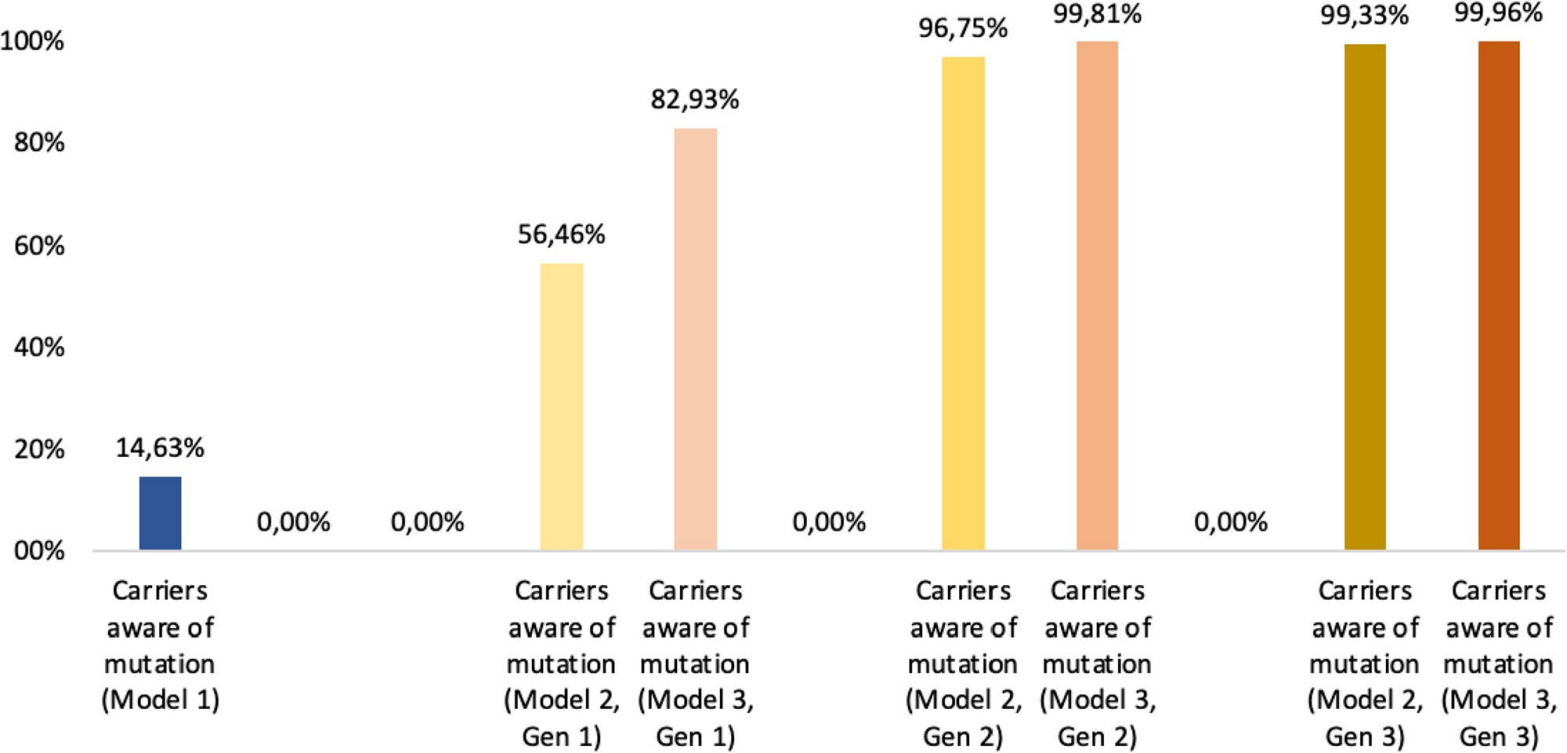
Carriers aware of BRCA 1/2 mutations.

**Figure 2:**
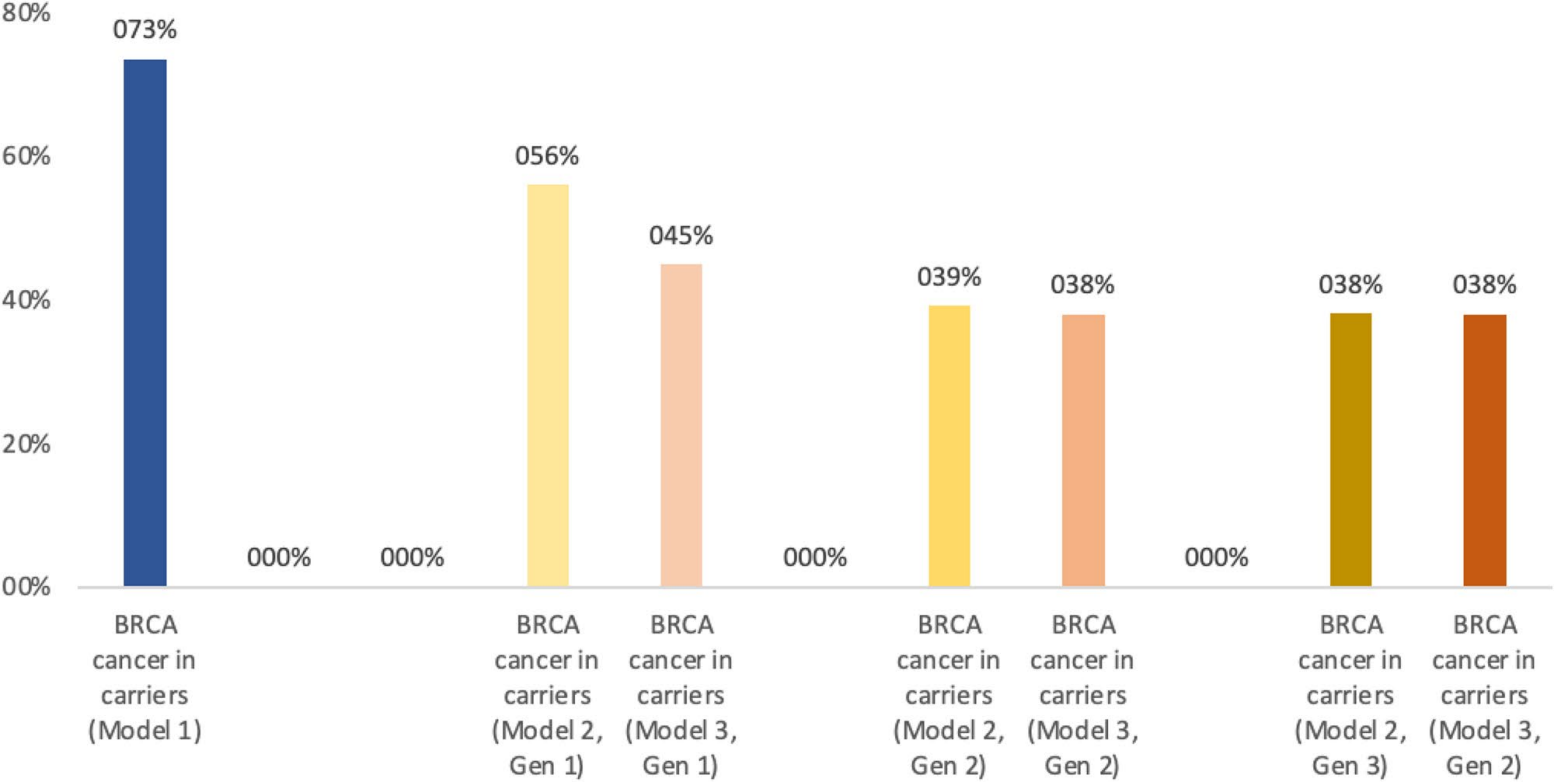
BRCA 1/2 related cancers in carriers.

The population not accessing genetic testing could either develop or *not* a BRCA-related gynaecologic cancer during lifetime due to a missed identification of the carrier status until cancer diagnosis. Two different approaches were designed and compared to select women for genetic testing:

- testing women who meet clinical criteria and/or have family history suggestive of BRCA gene carrier according to current recommendations^6,20–24^ (“*model 1*”, standard of care, Fig. 1);
- testing women who either meet criteria included in model 1 and/or are classified as having a high probability to be BRCA1/2 PV carriers according to a US-based radiogenomics model which is offered to women from the age of 18 years as part of routine gynaecological assessment (“*model 2*”, Fig. 2). The model was built on a multivariable analysis for classification of germline BRCA status via logistic regression, support vector machine, ensemble of decision trees and automated machine learning pipelines^16^. Moreover, an improvement of the performances of the radiogenomic model included in this model was simulated, reaching 80% sensitivity and 95% specificity (“*model 3*”). Enhancing model performance primarily involves implementing contrast adjustment techniques like histogram equalization, correcting intensity non-uniformity using methods such as N4 bias field correction, and applying noise filtering such as Gaussian blurring to minimize random variability^25–27^. Additionally, if necessary, auto-encoders based on convolutional neural networks can be employed to further improve the quality of images^28,29^. It’s worth noting that a multicenter international trial is currently underway with the specific goal of achieving these improvements (NCT05769517, ID PROBE II study).

All models were trained with literature data summarized in Table 1. Based on the confidence intervals of values reported in the literature, best-case and worst-case scenarios were defined, worst being the scenario with the highest prevalence of BRCA1/2 PV and related cancers.

**Table 1:** Italian data: incidence and direct costs of the NHS of BRCA mutated population^31^.

Data	Average	Best case	Worst case	
BRCA1/2PV carriers in gen. pop.	0.67%	0.59%	0.77%	^3^
Test eligible in pop gen (clinical criteria/family history)	0.98%	0.47%	1.79%	^11^, ABCFS
Test positivity probability	10.00%	10.00%	10.00%	^6,7,11^
BRCA1/2PV carriers in not eligible people	0.58%	0.51%	0.68%	^3^, ABCFS
RRSO and RRM in carriers	25.85%	35.84%	15.30%	^25–27^
RRSO only in carriers	29.15%	28.16%	29.70%	^25–27^
RRM only in carriers	21.15%	20.16%	18.70%	^25–27^
No prophylactic surgery in carriers	23.85%	15.84%	36.30%	^25–27^
Both breast and ovarian cancer risk in carriers	21.50%	14.81%	30.42%	^2^
Only ovarian cancer risk in carriers	9.00%	8.70%	8.58%	^2^
Only breast cancer risk in carriers	49.00%	48.20%	47.58%	^2^
No cancer probability in cancers	20.50%	28.31%	13.42%	^2^
Post RRM: only breast cancer	4.41%	0.96%	18.08%	^5^
Post RRM: only ovarian cancer	28.56%	23.20%	27.44%	^5^
Post RRM: both breast and ovarian cancer	1.94%	0.30%	11.56%	^5^
Post RRM: no cancer	65.09%	75.54%	42.92%	^5^
Post RRSO: only breast cancer	34.13%	23.09%	46.75%	^4^
Post RRSO: only ovarian cancer	0.80%	0.72%	3.85%	^4^
Post RRSO: both breast and ovarian cancer	0.42%	0.22%	3.95%	^4^
Post RRSO: no cancer	64.65%	75.97%	45.45%	^4^
Post RRM and RRSO: only breast cancer	3.49%	0.62%	15.82%	^4,5^
Post RRM and RRSO: only ovarian cancer	1.18%	0.93%	6.46%	^4,5^
Post RRM and RRSO: both breast and ovarian cancer	0.04%	0.01%	1.34%	^4,5^
Post RRM and RRSO: no cancer	95.29%	98.44%	76.38%	^4,5^
Cost				
Gynaecological visit and gynaecological US	51.65 €	51.65 €	51.65 €	^30^
Genetic counselling and genetic testing (6blocks)	1099,48 €	1099.48 €	1099.48 €	^30^
RRSO: DRG 359	1436.00 €	1436.00 €	1436.00 €	^31^
RRM: DRG 261	1960.00 €	1960.00 €	1960.00 €	^31^
Salpingo-Oophorectomy for ovarian K: DRG 357	6791.00 €	6791.00 €	6791.00 €	^31^
Mastectomy for breast K: DRG 258–257	3524.45 €	3524.45 €	3524.45 €	^31^
Chemotherapy	2226.00 €	2226.00 €	2226.00 €	^30^
PARP inhibitor therapy	82,344.00 €	82,344.00 €	82,344.00 €	^41^
Radiotherapy	6000.00 €	6000.00 €	6000.00 €	^30^
Yearly follow-up BRCA+	198.20 €	198.20 €	198.20 €	^24,30^
Yearly follow-up BRCA−/unknown	69.60 €	69.60 €	69.60 €	^19,30^
Age of consent: tests	18	18	18	
Average age of BRCA+ cancer onset	45	60	35	^38–41^
Average number of years in FU: STEP 1	27	42	17	
Average number of years in FU: STEP 2	35	20	45	
Total follow up	62	62	62	
Model exit age	80	80	80	
Expected age of prophylactic surgery	40	45	35	^18,39^

*ABCFS* Australian Breast Cancer Family Study.

Thresholds considered rely on those established for the use of diagnostic tests in clinical practice on the experience from Covid-19 pandemic^17^.

### Model input

Table 1 summarizes the key model inputs with their values, ranges and sources.

The probability of finding BRCA1/2 PV carriers among women selected according to clinical criteria and/or family history was set at 10% according to available evidence^7,12^.

The radiogenomic tool in *model 2* had a 49% sensitivity and 87% specificity^16^, while the one in model 3 had a 80% sensitivity and 95% specificity.

The probability of choosing prophylactic surgery in BRCA1/2 PV carriers was calculated according to published data^30,31^: RRSO only (29.15%), RRM only (21.15%), RRSO and RRM (25.85%), no prophylactic surgery (23.85%).

The risk of the occurrence of BRCA-related gynaecologic cancers in BRCA1/2 PV carriers was calculated also taking into account the impact of prophylactic surgery as cancer risk reduction factor^2,4,5,30^. Timing of life milestones in BRCA1/2 PV carriers was partly inferred from literature data and partly from available recommendations^30,31^.

Direct costs were considered applying the rates currently in use in Italy^32–34^. Direct costs incurred by Italian National Health Service (Servizio Sanitario Nazionale, SSN) for BRCA carriers identification and management included counselling and genetic testing, any prophylactic surgery (i.e. breast, uterine adnexa or both), treatment in case of onset of BRCA-related gynaecological cancers (i.e. breast, ovarian or both) and follow-up of both carriers and gynaecological cancer patients.

The total timeframe of the model was 62 years, considering as extremes the age of 18 years (the attainment of the age to express consent to perform a genetic test) and 80 years^2^.

### Analysis

A simulation of the impact of the introduction of radiogenomic screening for BRCA1/2 PV carrier status (*model 2*) in the generation of 18-year-old girls in Italy (274,000 people) was conducted^35^.

The number of incident gynaecologic-BRCA-related cancer cases in the reference population and the number of BRCA-related gynaecologic cancer-free life years (BRCA1/2 PV-HLYs) over a 80-year life span were considered as endpoints^36,37^.

Differences between *models 1* and *2* were analysed in terms of both costs incurred and BRCA1/2 PV-HLYs gained over the study time interval. An incremental cost-effectiveness ratio (ICER) was then derived to determine the cost in the population in order to increase BRCA carriers-healthy life span by 1 year.

Three subsequent generations were considered in the analysis. To assess the impact across generations, model 2 was advanced for two generations and then substituted by model 1 in the third one.

The analysis’ details and relative assumptions are reported in the Supplementary Materials.

## Results

Results of the models are reported in Tables 2 and 3, and Figs. 1 and 2.

**Table 2:** Results of the three models.

Population: 274.000; BRCA1/2PV: 1.836							
		Sensivity 47%–Specificity 87%			Sensivity 80%–Specificity 95%		
	MODEL 1	MODEL 2 Gen 1	MODEL 2 Gen 2	MODEL 2 Gen 3	MODEL 3 Gen 1	MODEL 3 Gen 2	MODEL 3 Gen 3
Eligible for genetic testing	2.685	37.650	37.373	1.841	15.614	15.525	1.836
Carriers detected	269	1.036	1.776	1.824	1.522	1.832	1.835
Carriers aware of mutation: cancer	102	392	673	690	576	694	695
Brca unknown: cancer	1.246	635	47	10	249	3	1
Total BRCA1/2PV cancer	1.348	1.028	720	700	826	697	695
Total BRCA1/2PV DALY	47.167	35.976	25.197	24.507	28.896	24.379	24.339
Total direct cost per 62 years-cycle (billions of €)	1.25	1.30	1.29	1.23	1.27	1.26	1.23
Cost/year/person (62 years; 274,000 people)	73.80 €	76.31 €	75.72 €	72.55 €	74.5 €	74.26 €	72.54 €

*Gen* Generation.

**Table 3:** ICER Model 2/3 vs Model 1 across three generations.

	Central scenario	Best-case scenario	Worst-case scenario
ICER GEN 1			
MODEL 2	3800 €	8097 €	2078 €
MODEL 3	653 €	1891 €	130 €
ICER GEN 2			
MODEL 2	1480 €	3380 €	922 €
MODEL 3	341 €	1281 €	− 55 €
ICER GEN 3			
MODEL 2	− 941 €	− 934 €	− 1210 €
MODEL 3	− 941 €	− 936 €	− 1210 €

*Gen* generation.

### Model 2

BRCA testing resulted in an incremental cost of 2.51 €/Year/Person for the first generation, compared to *model 1*, with an ICER of 3799.98 €. For the second generation, 1.92 €/Year/Person incremental cost was estimated, compared to model 1, with an ICER of 1480.18 €. A decremental cost of 1.25 €/Year/Person was estimated for the third generation, compared to *model 1*, with an ICER of − 940.94 €.

Compared to *model 1*, a 1300% increase of women eligible for genetic testing in the first generation was observed (37,650 vs 2685 women). Carriers identified before the onset of cancer were approximately 1036 (900 due to radiogenomic screening and 136 according to clinical criteria) vs 269 in *model 1*.

Considering RRSO and RRM options, the total number of BRCA-related cancers cases dropped by 24% compared to *model 1* (1028 women, of whom 635 unaware and 392 aware of the genetic carrier condition, vs 1348 of whom 1246 unaware of being carriers and 102 aware). Overall, by the end of model 2 first cycle, the total number of aware carriers increased to 1672 (91%) out of a total of 1836 carriers estimated based on epidemiological data. In *model 1* the estimated carriers aware of the condition at the end of the first cycle were approximately 1.515 but only 269 had been identified before the onset of cancer.

For the second generation, there was an estimated total number of 720 BRCA-related cancers (− 47% compared to the model 1). By the end of *model 2* s cycle, the total number of aware carriers increased to 1823 (99.3%). For the third generation, the radiogenomic mass screening tool was no longer included in patients’ selection and almost all carriers accessed genetic testing due to the anamnestic criterion alone. By adopting the appropriate risk reduction strategies, the number of BRCA-related gynaecological cancers dropped to 700 (− 48% compared to the initial scenario): this reduction from the *model 1* represents a threshold cut-off with respect to the potential for prophylactic surgery, given its low acceptance rates^30,31^ and the current performance of the screening model.

### Model 3

In this model, incremental cost resulted in 0.70 €/Year/Person for the first generation, with an ICER of 652.62 €, referred to the *model 1*; 0.46 €/Year/Person for the second generation with an ICER of 340.56 € and finally a decremental cost of 1.26 €/Year/Person was calculated for the third generation, with an ICER of − 940.90 €.

Compared to the *model 1*, a 482% increase of women eligible for genetic testing in the first generation was observed (15.614 vs 2.685 women). In the first generation, the number of BRCA-related cancers dropped by 39% (826 BRCA-cancer vs 1.348 in the *model 1*). Carriers identified at the end of the cycle were 1772, leading to a 96.5% rate of awareness of the genetic condition.

For the second generation, a total number of 697 BRCA-related cancers was reported (− 48% compared to *model 1*). The total number of people aware of the mutation at the end of the second cycle increased to 1835 (99.9%).

For the third generation, the mass screening model was no longer included in the selection process and almost all carriers accessed the genetic test thanks to the anamnestic criterion alone. By adopting the appropriate risk reduction strategies, the number of BRCA-related gynaecological cancers dropped to 695 (− 48% compared to the initial scenario). This reduction from *model 1* represents a threshold cut-off as already mentioned for the *model 2.*

## Discussion

This cost-effective analysis demonstrates that BRCA testing in women selected according to an integrated clinical-radiogenomic model could potentially lead to a significant reduction in BRCA-related cancers. In addition, it increases significantly the awareness of being a carrier, with a limited cost increase for the first two generations and a cost saving for the third one. The low prevalence of BRCA 1/2 PV makes its detection of a rather low impact on healthy life expectancy of the standard population. Nevertheless, the implications across generations, the availability of risk reducing strategies and the high costs related to ovarian and/or breast cancer management make the identification of carriers a real must.

The prevention of future cancers offsets the additional costs estimated in the first and second generation which are mainly related to the higher number of patients referred to genetic testing and therefore to prophylactic surgeries. In this context, the real value of an improvement of the performances of the radiogenomic model relies in the number of women referred to genetic testing (38,000 vs 16,000, first generation) corresponding to a saving of 24,178,000 €.

Despite the higher identification rate of BRCA1/2 PV carriers before the cancer onset related to the implementation of the mass radiogenomic screening, the effect in preventing BRCA-related cancers is limited. The current estimated rate of acceptance is lower than 30%^30,31^, thus reducing the clinical benefit of early BRCA carriers identification. For the mass screening approach to be effective, the offer of risk reducing surgeries for BRCA carriers should be better supported by policy-makers, public health professionals and clinicians. Nevertheless, given the reproductive implications of RRSO, it has to be acknowledged that there will always be women who do not wish to pursue or would rather delay RRSO, hopefully benefitting from enhanced cancer surveillance. It is highly likely that sequencing costs will further plummet in the next few years, thus reducing the economic impact of a much broader offer of testing due to the mass radiogenomic screening.

Our study has certain limitations. The radiomics model awaits validation in external cohorts and is constrained by data gathered from a referral center, which may not completely mirror real-world scenarios. The forthcoming results of the ongoing multicenter international trial, PROBE II, will play a pivotal role in advancing the generalizability of the outcomes.

The acceptance rates of risk-reducing surgeries are derived from existing literature data, which may not necessarily capture the characteristics of the broader population or current conditions.

The lack of indirect costs assessments is a limitation of our study and a possible development for future research. Although the payer perspective is used in some studies and in some countries, WHO and other authors recommend the adoption of a societal perspective, in which effects and costs of cancer are also estimated outside of health care (indirect impacts and indirect costs, including impacts on caregivers)^38^. Our model constitutes a theoretical model in which it has been deliberately chosen to ignore some factors such as reproductive choices, the adherence to counselling and subsequently to genetic testing, once the indication has been established by a specialist or by screening. In our model it has been assumed a total adherence to genetic testing (100%), to convey the potential effect of an extensive awareness of BRCA mutational status by the population. Available data show a rate of uptake of genetic testing of approximately 70%^38^. In addition, our models do not take into consideration the implications of BRCA1/2 variants of uncertain significance (around 2%)^39^.

The potential cost-effectiveness of BRCA testing implementation is consistent with other previous work^11,26,40^. Population-based BRCA testing in the Ashkenazi Jewish population is known to be feasible, acceptable and effective^41^. BRCA testing has been reported to be cost-effective and associated with reduced risk of cancer and improved survival. Indeed, searching for BRCA mutations in breast cancer patients results in an incremental cost per quality-adjusted life-year (QALY) gained of AU $18,900 with a 0.04 risk reduction of breast cancer and 0.01 risk reduction of ovarian cancer Subsequent testing of also family members results in an incremental cost per QALY gained of AU $9500 with an additional reduction of 0.06 of breast and 0.01 of ovarian cancer respectively^40^. Finally, a population-based testing implementation seems to be cost-saving and cost-effective in high income countries, cost-effective in upper middle income countries, but not cost-effective in low income countries^12^. Such approach could prevent 2319 to 2666 breast cancer and 327 to 449 ovarian cancers per million women.

## Conclusions

A radiogenomic US based model can refine the assessment of a priori probability to access BRCA testing and seems to be already an effective, sustainable strategy with long term benefit. The incremental costs per person-year for such strategy are low due to the rarity of the condition; the calculation of indirect costs of cancer as well as the physiological reduction of sequencing costs over time, are highly likely to further reduce such costs. Nevertheless, compliance and adherence to risk reducing strategies is crucial to offset the additional costs calculated, enhancing the benefits of such increased identification rate. Future studies are needed to improve the model performances in order to maximize the effect of cancer prevention/early detection already in the first generation.

### Supplementary Information

Supplementary Information 1.

Supplementary Information 2.

## Data Availability

All data generated or analysed during this study are included in this published article [and its Supplementary Information files].
