# Supplementary figures and images for: A cost-effectiveness analysis of an integrated clinical-radiogenomic screening program for the identification of BRCA 1/2 carriers (e-PROBE study)
Source: Sci Rep. 2024 Jan 9;14:928. doi: 10.1038/s41598-023-51031-1 (PMC10776619; doi:10.1038/s41598-023-51031-1)

FLOWCHART: MODEL 1

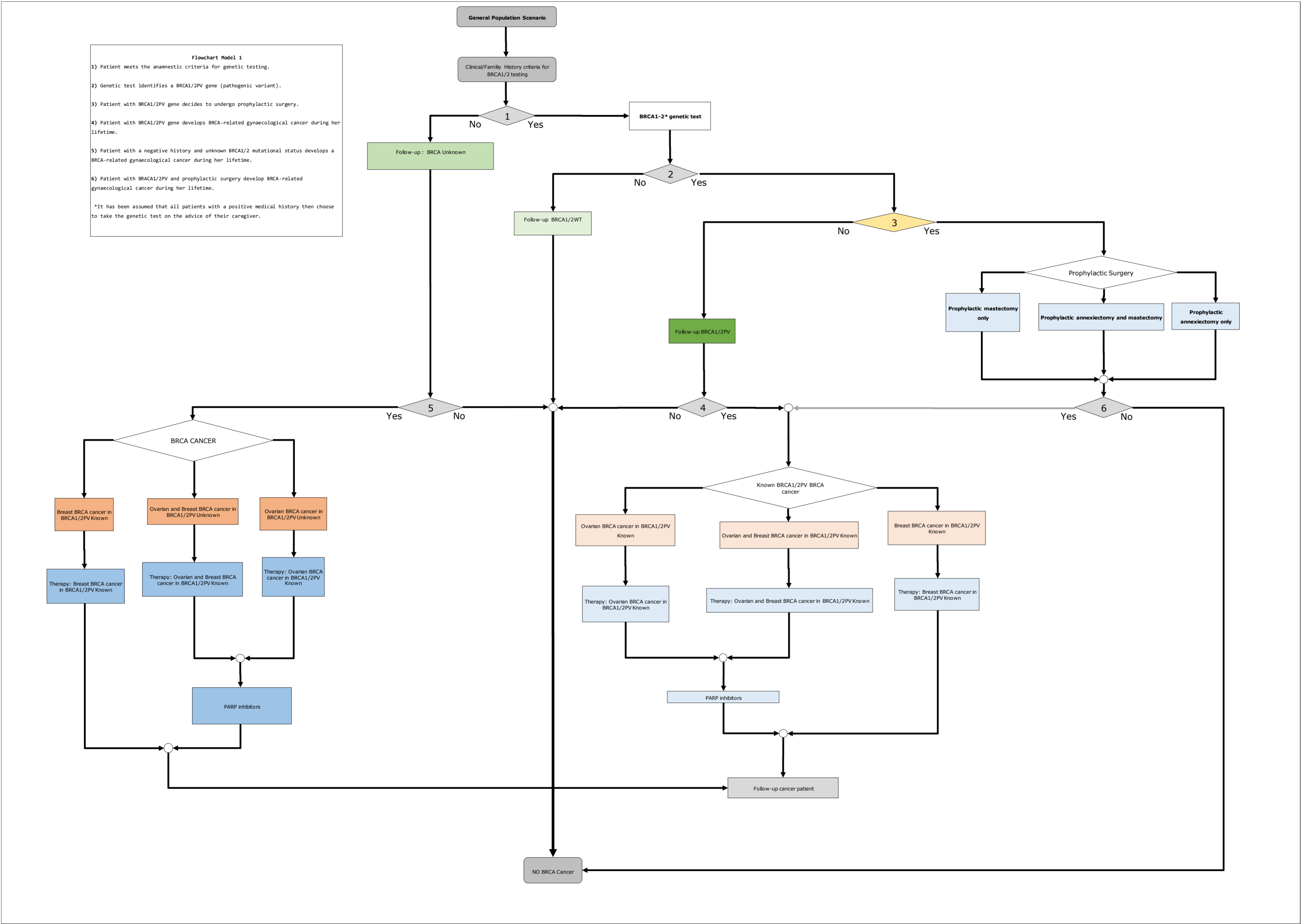

Supplement: Supplementary file 1 — Supplementary Information 1. [file 41598_2023_51031_MOESM1_ESM.pdf]

**FLOWCHART: MODEL 2/3**

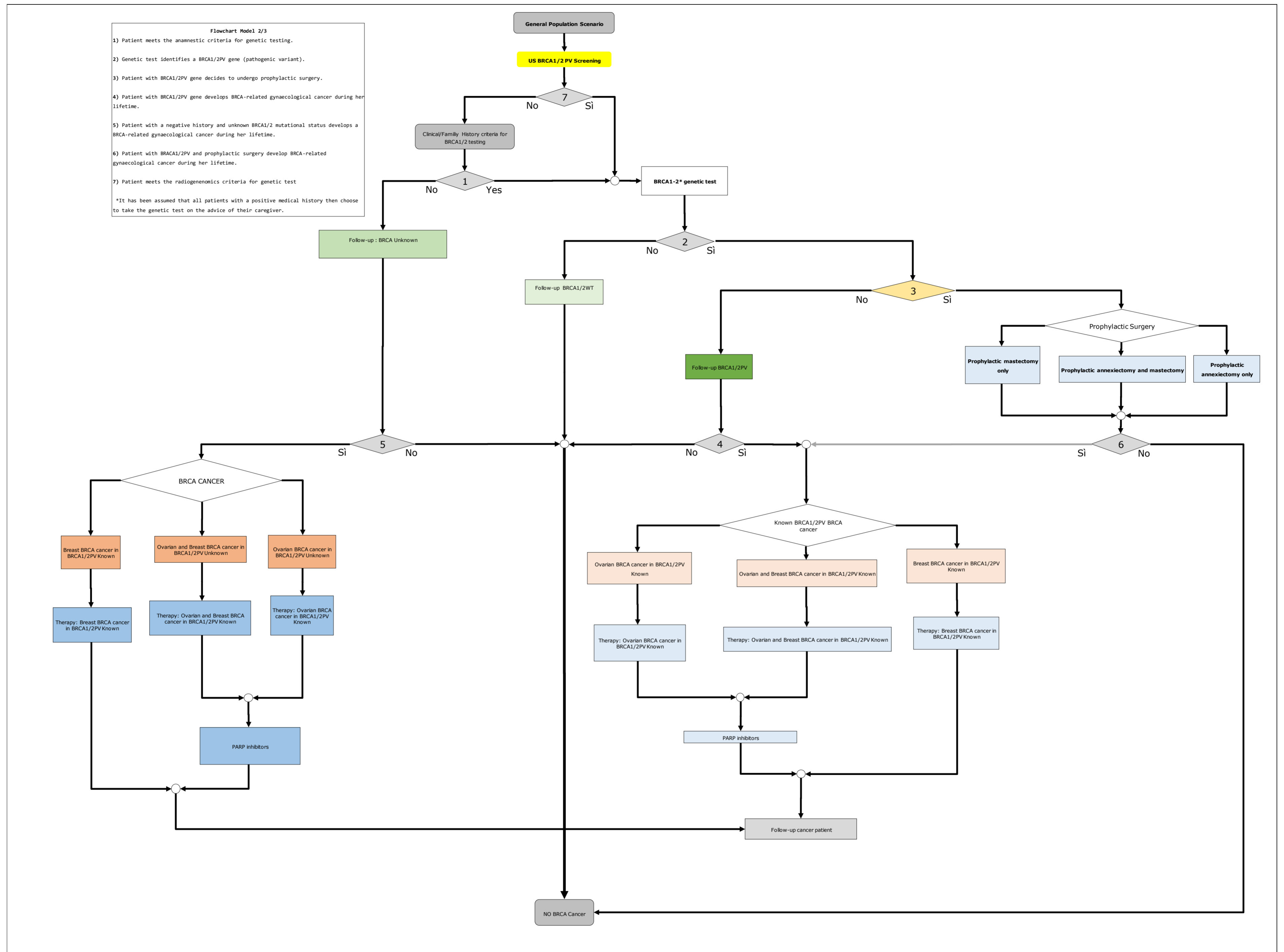

Supplement: Supplementary file 2 — Supplementary Information 2. [file 41598_2023_51031_MOESM2_ESM.pdf]
